# Removal of sulfate pollutant from different samples of a river water using nanozeolite technology, case study: Gamasiab River, Iran

**DOI:** 10.1371/journal.pone.0314480

**Published:** 2025-02-10

**Authors:** Amin Rezaei, Hossein Babazadeh, Amir Khosrojerdi, Mahdi Sarai-Tabrizi

**Affiliations:** Department of Water Science and Engineering, Science and Research Branch, Islamic Azad University, Tehran, Iran; Rajendra Memorial Research Institute of Medical Sciences, INDIA

## Abstract

Human activities significantly impact on river water quality as a crucial water source. A study in the Gamasiab River analyzed samples from 16 points at three time periods, assessing element concentrations. The most polluted station was identified using spectrophotometric testing and treated with natural and modified zeolite nanoparticles for purification. Various acid and base combinations modified the nanoparticles, optimizing their effectiveness as adsorbents through tests under different conditions. Utilizing the Design Expert model, theoretical adsorption values were determined based on pH and adsorbent-pollutant ratio. The modified samples demonstrated 77% efficiency with 0.2 molar nitric and sulfuric acid. Interaction studies showed how phosphate and nitrate ions affected sulfate adsorption. Optimal adsorption conditions were defined at pH = 9.6 and D/C = 17.01, achieving 86.5% pollutant adsorption. The Freundlich isotherm, with a coefficient of determination of 0.92, was chosen over the Langmuir isotherm (0.79) for its superior performance. Therefore, applying zeolite nanoparticles efficiently eliminated sulfate pollutants from surface water resources at the laboratory.

## Introduction

Accessing drinking water has always been one of the major challenges for human beings, and its availability is considered a significant obstacle in various societies [1]. Water resources may become polluted due to human activities, atmospheric conditions, and geological features. Sulfates are well-known pollutants in rivers, commonly found in regions with sedimentary rocks or near oil wells. Additionally, they can be present in citrus plantations and fields due to the usage of sulfate-containing fertilizers or effluents from some factories. Similar to other minerals, sulfate can form scale-like layers in water pipes, resulting in an unpleasant taste in water that is unsuitable for consumption by humans and domestic animals. Moreover, sulfates can hinder the effectiveness of washing clothes [2–4].

In the current century, there is an urgent need for technological innovation in integrated water management. Nanotechnology has great potential to advance this objective among existing technologies. By enhancing the efficiency of water and wastewater treatment, this technology contributes to the provision of clean and safe water by utilizing unconventional water sources [5]. Previous studies revealed toxic ions like copper, lead, nickel, and cadmium can be separated from aqueous solutions using porous silica nanoparticles [6], or lead, copper, and silver can be removed from water by using phosphorus nanofibers [7].

All these instances involve the use of natural nanoparticles to purify target ions from water solutions [8]. However, the most intriguing aspect of this technology is the utilization of natural nanoparticles to enhance the purification capabilities of nanoparticles and improve their efficiency in removing a wider range of pollutants. For example, domestic wastewater can be purified using iron nanoparticles synthesized from extracts of various plant leaves, offering an environmentally friendly solution [9], or employing natural zeolite and magnetic carbon nanotubes to effectively remove sulfate pollutants, a significant challenge in rivers near agricultural fields, from aqueous solutions [10]. Additionally, a new frontier in water purification science may be explored through the application of organic micro-polymers [11]. For instance, recent studies have demonstrated the effective removal of selenium from aqueous solutions using magnetic nanoparticles coated with hematite [12]. Furthermore, zirconium-based oxides have emerged as a promising choice for water treatment. These oxides offer several advantages, including high absorption capacity, exceptionally low water solubility, and outstanding selectivity for the adsorption of sulfate ions [13–15]. Under specific conditions, a saturated sulfate adsorption capacity of 284.22 mg/g was achieved, leading to only a 10% decrease in capacity even after five cycles. The mechanisms of sulfate adsorption and desorption are controlled by ion exchange with surface hydroxyl groups [16].

Previous studies have focused on the removal of dyes and heavy metals. However, the present study evaluated anionic and cationic pollutants. Furthermore, the optimal performance conditions of the adsorbents were modeled using the Design Expert model by calculating the optimal pH and the ratio of adsorbent to pollutant. The study also emphasizes the use of zeolite nanoparticles for purifying river water samples, various enhancement methods for improving the adsorbent, and its impact on different pollutants. Additionally, the study considers the interaction effects of these adsorbents, as well as other ions in water, and their influence on the adsorption capacity.

## Materials and methods

### Characteristics of the region under study

**Fig 1:**
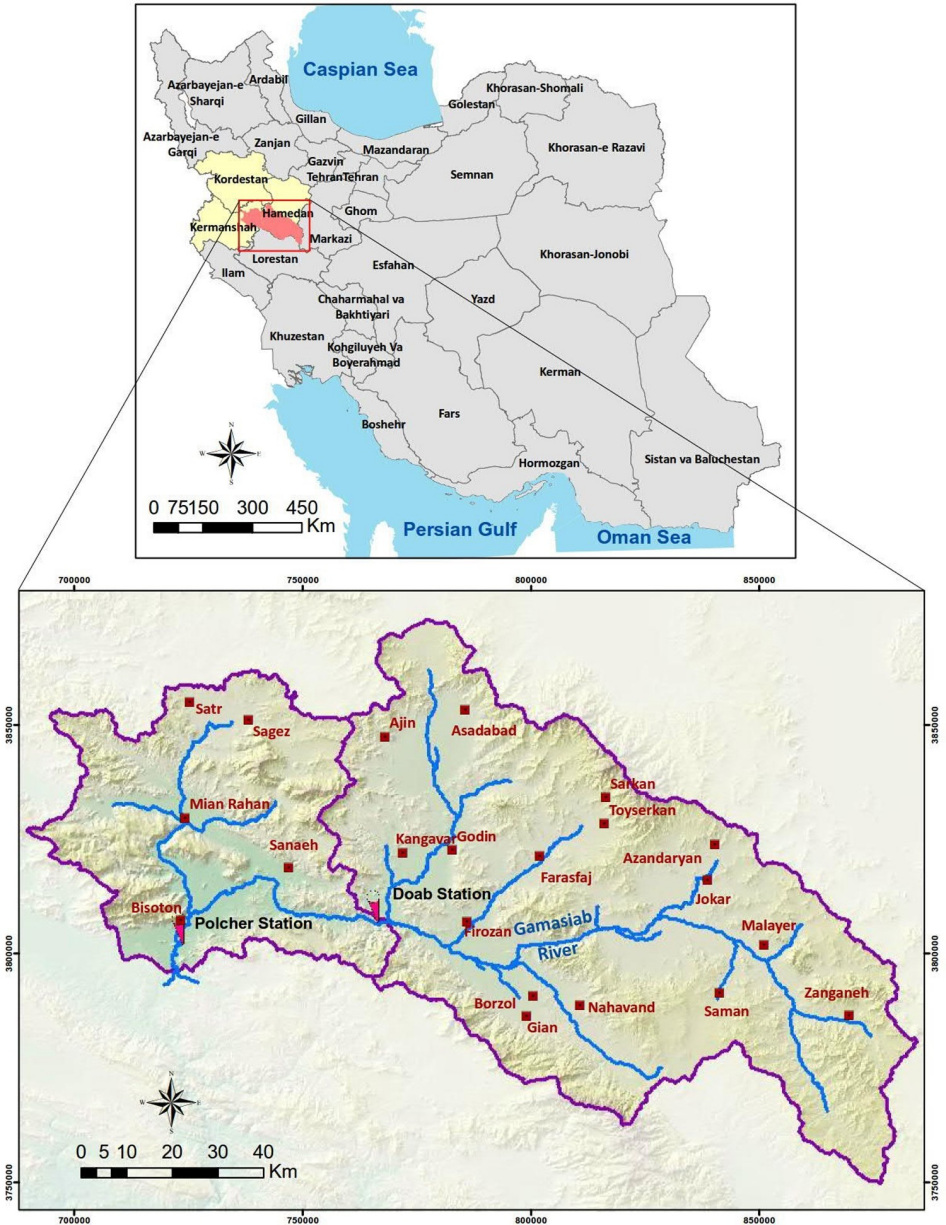
The studied river, stream gage location, and population points in the river basin (background terrain map was used from Natural Earth public domain http://www.naturalearthdata.com).

Gamasiab River is located in the eastern region of Kermanshah province. The geographical location Gamasiab River is between 47˚ 21ʹ-47˚ 54ʹ east and 34˚ 16–34˚ 53ʹ north. Fig 1 shows the study area of this river. The total length of the Gamasiab River is about 74.7 km. The flow of this river begins from Sarab Seng Soorakh and limestone springs in Gamasiab village located in the west of Hamedan province (northern slopes of Green highlands called Sarab Gamasiab). The water flow in the river is in the east to west and northwest to southwest direction, which joins to the water of Dinevar River. Here, two phases were considered to study the Gamasiab River. The first phase includes the area from the point where the river enters Kermanshah province to the confluence with the Dinevar River with a length of 4.34 km, and the second phase includes the area from that point to the confluence with the Qaresou River and the formation of the Seymareh River with a length of 3.40 km. Totally, 16 stations were used to sample river water in the study area.

### Sampling method and location

#### Selection criteria of sampling sites/stations

As the upstream of the important Karkheh River in Iran, the Gamasiab River plays a significant role in its water quality. Considering the conditions and characteristics of the target river, stations were used to sample the water quality after consulting with regional water experts. The above-mentioned locations were selected based on the condition of the river and its sub-branches. Then, two stations were specified for each sub-branch or water supply channel including one before and one after the main branch. Finally, the samples were examined based on pH, sulfate, nitrate, phosphate, and iron. Table 1 presents the coordinates and the reason for selecting each station.

**Table 1:** Sampling stations and geographical locations on Gamasiab River, Iran.

Station name	Longitude (degree, Minute, second)	Latitude (degree, Minute, second)	Reason for selection
1	47-39-19	34-21-43	Water quality measuring station
2	47-39-08	34-25-35	Downstream agricultural land
3	47-39-40	34-26-55	On the location of Darakeh diversion dam
4	47-39-03	34-27-40	Sahneh Springhead
5	47-35-18	34-27-08	At the location of the diversion dam reservoir
6	47-35-04	34-27-09	Downstream of Ahangaran village diversion dam
7	47-34-02	34-26-45	Location of the bridge
8	47-33-09	34-26-15	Ahangaran village drainage outlet
9	47-27-55	34-24-13	Measuring changes in the water quality of Gamasiab along the route
10	47-27-20	34-23-47	Proximity of the place where Dinourab is poured into Gamasiab to determine the water quality of Gamasiab before joining Dinourab
11	47-27-18	34-23-47	Determining the quality of Dinourab water before joining Gamasiab and after passing through Bistoun city
12	47-26-40	34-23-20	After the confluence of Gamasiab and Dinourab
13	47-26-26	34-22-33	Measuring the quality impact of water entering Bistoun springhead to Gamasiab
14	47-25-06	34-21-43	Assessing Bistoun industrial town, its refinery, and power plant on the water quality of Gamasiab
15	47-22-03	34-13-28	Measuring the effect of the vast agricultural complex along the river in this section on water quality
16	47-20-47	34-10-39	Evaluating the quality of Gamasiab water passing through Kermanshah at the end of its route

The water samples were poured into 90 ml volume plastic containers free from contamination and transferred to the laboratory in an insulated container to prevent temperature exchange.

#### Laboratory equipment

Here, the spectrophotometry device model DR5000 from the Hek Company was utilized to determine the concentrations of pollutants. Then, the pH of the samples was measured applying a pH meter model 713 from the Metrom Company. In addition, a scale of the model Electronic balance KEB5003 made in Switzerland with an accuracy of +/- 0.0001 was used for weighing the samples. Regarding the atmospheric creation of nitrogen for coating iron-containing compounds with nanotubes, a nitrogen capsule made in Iran named BM55E was employed. Further, FT-IR devices of the model Cary604 made by the Agilent America Company, X-ray diffraction (XRD) Xpertpro model made by Panalytical Company in the Netherlands, and TEM were utilized to determine the shape and size of zeolite nanoparticles. The study was conducted on organic compounds containing zeolite nanoparticles and their quality utilizing this equipment.

#### Method

In order to conduct the study, natural zeolite was prepared from the Isfahan, Iran mine, crushed by a hydraulic jack machine and passed through a sieve with 400 mesh. Then, zeolite nanoparticles with dimensions less than 100 nm were generated applying a ball mill machine. A certain amount of zeolite sample was added to 100 ml of water and stirred for two hours at a speed of 200 rpm at room temperature (25°C). The lids of the sample containers were fastened and kept statically in the environment for 24 hours. Then, the solution was passed through a filter paper and placed in an electric furnace at a temperature of 100°C for 24 hours [17]. The results of XRD, FT-IR, and TEM devices were employed for determining the quantitative and qualitative characteristics of zeolite nanoparticles.

The samples collected from the river were measured for sulfate after being transferred to the laboratory based on the standard method [18] using a spectrophotometric device and a sulfate detector kit. The kit included barium chloride and citric acid as stabilizers. After transferring the sample to the 10 ml tube of the device, the indicator was added to the sample and stirred until it was completely dissolved. Then, the sample was rested for 5 minutes, and the machine was cleaned. To determine the sulfate concentration, the cell was cleaned and placed in the chamber of the device, which was set to a wavelength of 450 nm.

After measuring the amount of sulfate, the samples obtained from the measurement during August were selected as the most polluted due to the decrease in the river discharge and the use of sulfate-containing fertilizers by the farmers. In the next procedure, zeolite nanoparticles were added to the target samples for sulfate purification through a surface absorption mechanism. It is noteworthy that at this stage, the pH of the samples was considered as the pH of the sample taken from the river, and the ratio of adsorbent to the pollutant at a temperature of 25°C for each adsorbent was equal to 10 grams of adsorbent to 90 ml of solution, which was 100 mg/liter. The purification results were investigated by a spectrophotometer after the desorption of nanostructures. In the next step, the samples were tested for sulfate in certain time intervals (included 0.5, 1, 6, 12, 24, and 48 hours after applying the adsorbents) to find the adsorption equilibrium time after applying the adsorbent. During the aforementioned time intervals, the efficiency was calculated based on Eq (1) every time after the desorption of the nanostructure and measurement of sulfate remaining in the sample.

$$\text{Removal}=\frac{{\text{q}}_{\text{i}}-{\text{q}}_{\text{t}}}{{\text{q}}_{\text{i}}}$$ (1)

where q, q_i_, q_t_, and Removal represent the concentration, initial concentration of the pollutant, concentration of the pollutant after treatment, and absorption percentage, respectively.

#### Modifying zeolite adsorbent

A certain amount of clinoptilolite zeolite was poured into 100 ml of water and stirred for 2 hours at a speed of 200 rpm at a temperature of 25°C. Then, the lid of the container was closed and left for 24 hours. The resident was kept in the environment. The samples were passed through filter paper and placed in an electric oven at 100°C for 24 hours. The dried material was added to 100 ml of distilled water immediately after leaving the oven. In the next step, 10 ml of a modification or combination solution was added [2]. To avoid changing the pH of the existing solutions with the modified samples, they were washed again with distilled water and dried [19]. The significant issue in the modification methods is that the samples which are dried in room air lose their properties and fail to perform properly due to the oxidization of zeolite by oxygen and moisture. To eliminate this obstacle, nitrogen gas was constantly blown in the furnace with a certain flow rate during the treatment of zeolite with modifiers [18].

Acid modification opens the pores of zeolite, leading to a better absorption process. In most of the cases, strong acids such as hydrochloric acid, sulfuric acid, and nitric acid yield better results than other modifiers [20,21]. Furthermore, zeolite loses some of its properties in modification with basic and alkaline solutions since it oxidizes during drying in open air [22]. To reduce the repetition of the names of samples modified by different methods in the study, a name was selected for each zeolite modification method (Table 2).

**Table 2:** Adsorbents modified using different solutions.

Adsorbent	Type of modification	Drying method
Z1	Natural zeolite	Air proximity
Z2	1% molar of nitric and sulfuric acid	Air proximity
Z3	2% molar of nitric and sulfuric acid	Air proximity
Z4	1% molar of nitric and sulfuric acid	Blowing nitrogen gas
Z5	2% molar of nitric and sulfuric acid	Blowing nitrogen gas
Z6	1% M aluminum chloride	Air proximity
Z7	1% M aluminum chloride	Blowing nitrogen gas
Z8	1% M of salicylic acid	Air proximity
Z9	1% M of salicylic acid	Blowing nitrogen gas
Z10	1% M ammonia	Air proximity
Z11	1% M ammonia	Blowing nitrogen gas

#### Eliminating the interaction of pollutants

To investigate the effect of interfering ions on sulfate removal, experiments were conducted in the presence of 5 mg/liter of sulfate ions and varying concentrations of symbiotic phosphate ions [23]. Phosphate ion concentrations ranging from 100 to 500 mg/liter (100, 200, 300, and 500 mg/liter) were introduced into the solution, and the absorption process was repeated. The pH of the solution was maintained equivalent to that of the river water sample. The aforementioned experiment was replicated with the specified values and conditions for sulfate ions, as well as with different concentrations of nitrate and iron. However, this method did not account for the impact of other ions and substances soluble in water.

#### Designing the experiment and design expert model

The Response Surface Methodology (RSM) was employed to design the experiments in this study. The RSM is considered as one of the optimization methods which models obstacles using mathematical and statistical techniques. The aforementioned method reduces costly simulation runs, as well as predicting the natural procedure of process optimization, which is often non-linear. Response surface methodologies can be designed in different ways, depending on their use in the experimental design such as CCD, D-Optimal, and Box-Behnken methods. The surface methodology has extensive application in industries including the chemical, petrochemical, fisheries, and microbiology industries [24].

A cost-effective method should be used for obtaining the maximum performance of the nanostructure. The central composite design (CCD) was the most important and comprehensive method among surface response methodologies. Therefore, the CCD was selected to evaluate two independent variables accurately. The ratio of the adsorbent (in mg/L) to the initial concentration of the pollutant (in mg/L) and pH were selected as control variables. The above-mentioned variables were selected due to the availability of the information needed. Designing the experiment included a full factorial in two levels (2^3^ = 8), four-star points, and one central point. In addition, three replicate experiments were performed at the central point to assess the net error between each experiment. In designing the experiment, no non-linear relationship is observed between variables when two levels are considered for each parameter. Thus, at least three levels are needed to show the non-linear behavior of independent variables [24].

The Design Expert model is a statistical model presented by Stat-Ease (1988) specifically for designing experiments. This model can be utilized for performing comparative tests, screening, and mixing plans. The Design Expert model provides a test matrix for screening up to 50 factors. The statistical significance of these factors was established by ANOVA [25].

The steps of the aforementioned process are as follows.

*Objective determination*: First, the objectives of the experiment are defined. In this regard, the response variables (outputs) we want to optimize or better understand are specified. These variables can be factors such as product quality, production efficiency, or any other measurable output.

*Identifying factors and levels*: The factors or variables which may affect the response variables are determined. These factors can be controllable like temperature or pressure, or uncontrollable such as environmental conditions. Finally, the levels or settings at which we want to test these factors are specified.

*Experiment design*: The Design Expert provides tools for creating an appropriate experiment design, often based on the RSM. This design involves selecting specific combinations of the levels of surfaces to run the experiments to obtain sufficient data points and develop a mathematical model for describing the relationship between factors and responses.

*Performing tests*: The tests are run according to the designed schedule. The values of the response variables are recorded for each combination of factor surfaces.

*Data Analysis*: Design Expert helps analyze test data, as well as using statistical techniques to fit response surface models. These models provide equations which describe how factors affect responses. Such equations are used to predict and optimize the process.

*Optimization*: After obtaining the response surface models, the software is used for finding the optimal settings of the factors, which involves maximizing or minimizing response variables to achieve the objectives. The software often provides graphical tools to visualize response surfaces and help identify optimal conditions.

*Confirmation*: Confirming the optimal conditions involves running additional tests to ensure that the predicted results match the actual ones in real-world conditions.

*Formula generation*: In Design Expert, the software may provide mathematical equations representing the relationship between factors and responses. These equations are used for further analysis and decision-making.

*Repeat as necessary*: Depending on the results, you may need to repeat the process and make changes to factors or levels to optimize your process.

Based on the limitations in laboratory conditions, the samples were simulated for pH and ratio of adsorbent to different pollutants using the Design Expert model. In order to investigate the effect of sulfate removal efficiency by nanozeolite adsorbent, two factors including pH and D/C were selected in the range of 3–14 and 5–100, respectively.

#### Adsorption isotherm

Isotherm is considered as the most important parameter in designing adsorption systems, which describes the relationship between the concentration of the adsorbed material and the adsorption capacity of an adsorbent. In this regard, the two existing isotherms including Langmuir and Freundlech were studied. The Langmuir isotherm is estimated based on the assumption of valence points on the adsorbent surface. Therefore, the adsorbed layer is considered as one molecule thick [26]. The linear Langmuir equation is estimated as follows.

$$\frac{C_{e}}{q_{m}}+\frac{1}{K_{l}*q_{m}}-\frac{C_{e}}{q_{e}}$$ (2)

where C_e_ shows the equilibrium concentration of the substance in the solution, mg/L, q_e_ indicates the amount of substance adsorbed per solid weight of substance mg/g, K_l_ is considered as the Langmuir coefficient, and q_m_ is regarded as the maximum theoretical adsorption rate of the Langmuir model. The Langmuir adsorption model index is calculated by Eq (3) where C_e_ is considered as the maximum initial concentration, mg/L, K_l_ shows the Langmuir constant number, and R_l_ indicates the Langmuir index. In Eq (3), R_l_ = 0 is an irreversible model, R_l_ = 1 is a linear model, and 0<R_l_<1 is the favorable model when R_l_>1 is an unfavorable model.


$$R_{l}=\frac{1}{1+K_{l}*C_{0}}$$ (3)

**Fig 2:**
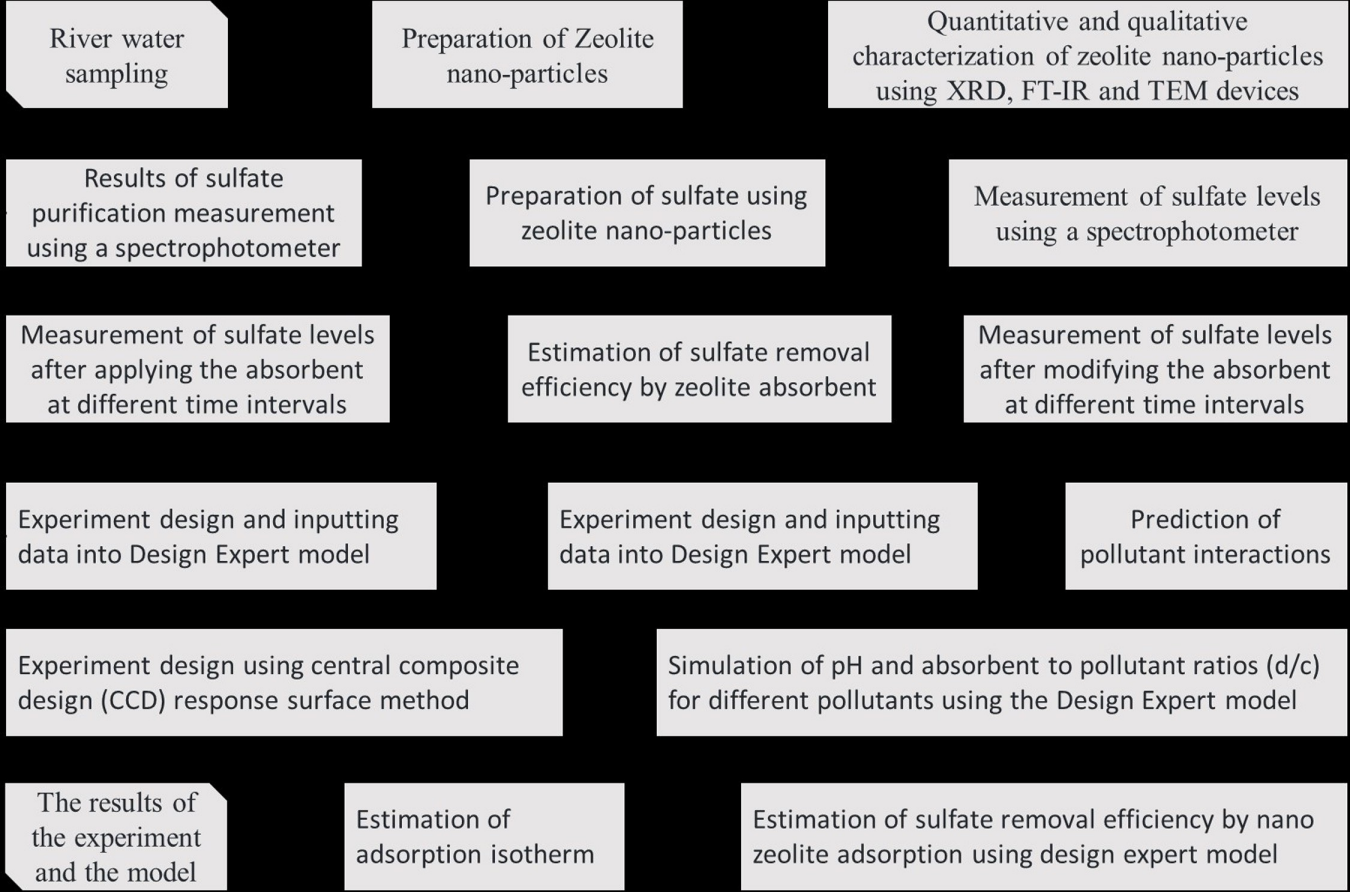
Process overview of nano zeolite purification test process and design expert model.

The multi-layer adsorption isotherm is expressed by the Freundlich model, which shows that the adsorption areas on the surface are not the same and have different adsorption powers. The Freundlich linear equation is usually defined as Eq (4) where q_e_ indicates the equilibrium concentration of the adsorbed ion in mg/g, C_e_ is regarded as the equilibrium concentration of the pollutant in mg/L, and K_f_ and n are considered as the Freundlich absorption coefficient in L/mg. Therefore, the closeness of the coefficient of determination (R^2^) to one increases the closeness of the given isotherm to the results obtained from the laboratory. To find the best adsorption kinetics and isotherm, the test data are checked using RMSE and R^2^ statistical methods, and the adsorption kinetics and isotherm are selected after fitting the known kinetics and adsorption isotherm models (Ho, 2004). Fig 2 shows the nano zeolite purification test process and the Design Expert model.


$$\log\;q_{e}=\log\;k_{f}+\left(\frac{1}{n}\right)\log{\text{c}}_{\text{e}}$$ (4)

## Results

### Determining the amount of sulfate

The sampling was conducted from the specified stations on the Gamasiab River during three one-year time intervals and on the 0^th^ day of the three-time intervals, specifically in April (mid-period), August (drought period), and February (wet period). The parameters of phosphate, nitrate, iron, and sulfate were measured in the samples, and the results of the measurements were expressed in the following figures.

The results indicated that the amounts of phosphate, nitrate, iron, and sulfate parameters varied during three periods. The amounts of phosphate and nitrate were normal, while the amounts of iron and sulfate were at the optimal level in the mid-period (May). The amount of phosphate and nitrate decreased, while the amount of iron and sulfate increased during the drought period (August). The phosphate and nitrate values improved, while the iron and sulfate values returned to optimal levels in the wet period (February). Based on the results, the pollution level of the Gamasiab River is varied and affected by different factors such as season, drought, and wet periods during three different time intervals. The amount of phosphate and nitrate was normal, while the amount of iron and sulfate was optimal in the mid-period (May). However, during the drought period (August), the amount of phosphate and nitrate decreased, while the amount of iron and sulfate increased, which may be related to the lack of water and the decrease in river flow. The amount of phosphate and nitrate improved, while the amount of iron and sulfate returned to the optimal level in the wet period (February).

**Fig 3:**
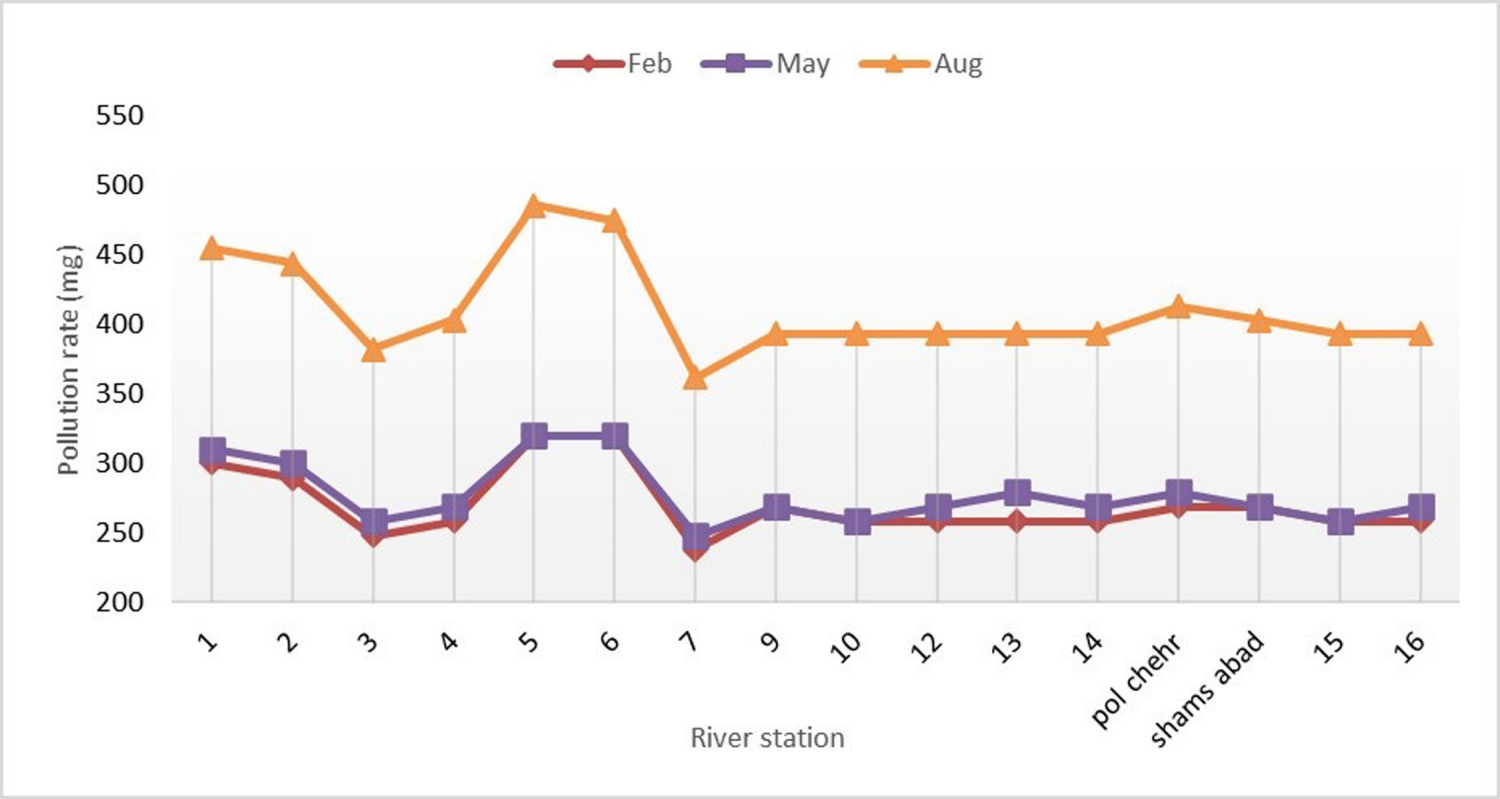
Changes of sulfate along the Gamasiab river.

As displayed in Fig 3, which shows the stress created by sulfate changes, the stress occurs due to the construction activity in stations 5 and 6. At this site, a construction site was established to build the bridge. Construction activities increase during the summer due to the decrease in river water flow and the cessation of rainfall, leading to the growth of sulfate stress. In addition, using sulfate-containing fertilizers in fields and gardens near the river in summer raises sulfate pollution in the river water.

Accordingly, the changes in sulfate in the river depend on several factors including construction activities, reduction of river water flow, cessation of rainfall, and use of sulfate fertilizers.

**Fig 4:**
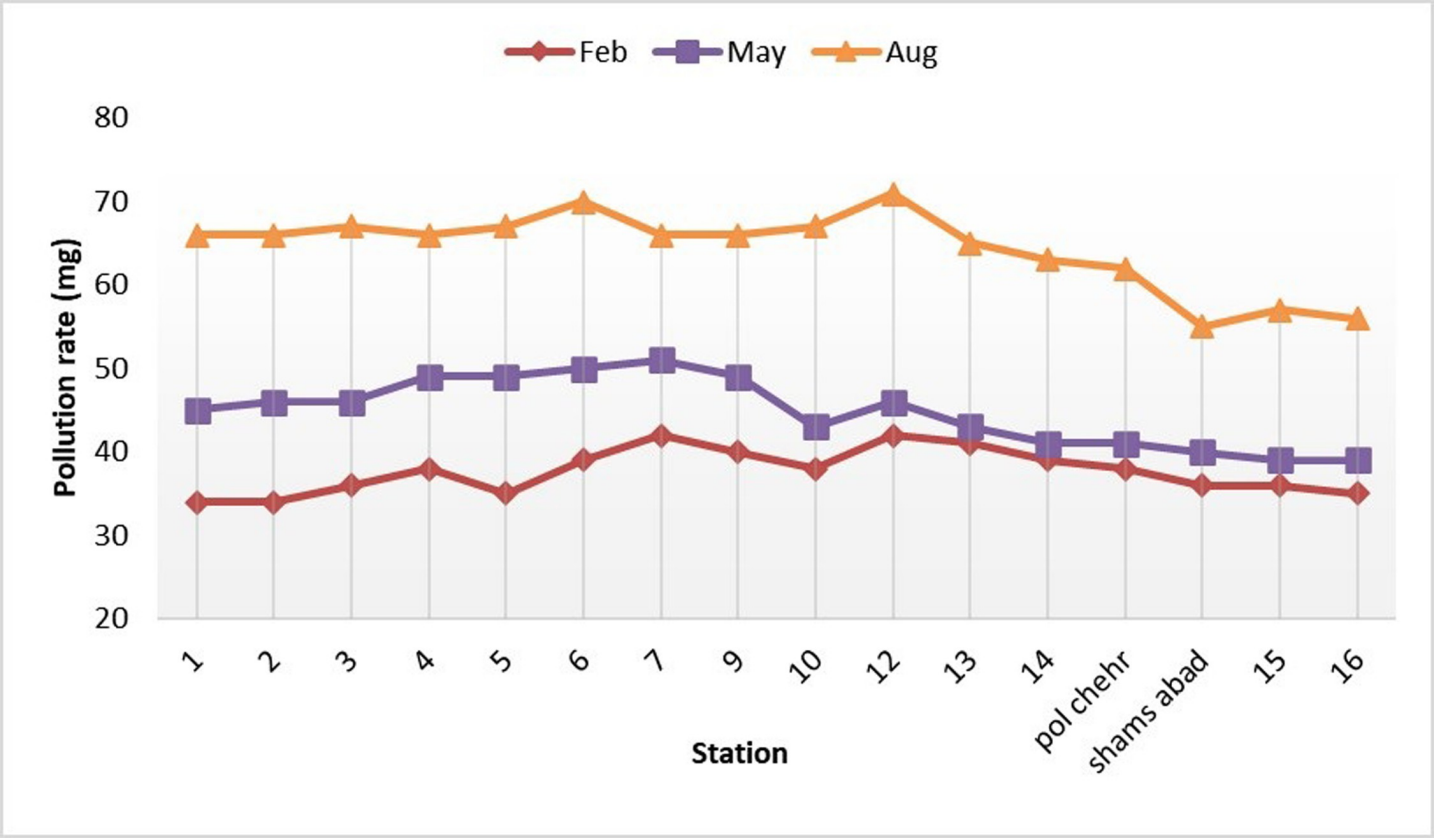
Changes of nitrate along the Gamasiab river.

Fig 4 shows the changes in nitrate parameter along the river in three time-intervals. As shown, there are maximum and wash points during different seasons due to the river water passing through agricultural fields in this area, which depend highly on nitrogen-containing fertilizers. Generally, the values of nitrate parameter increased significantly following the reduction of the river water flow and the cessation of rainfall during the summer. The nitrate parameter changes in the river depend on several factors including agricultural activities and use of nitrogen-containing fertilizers.

**Fig 5:**
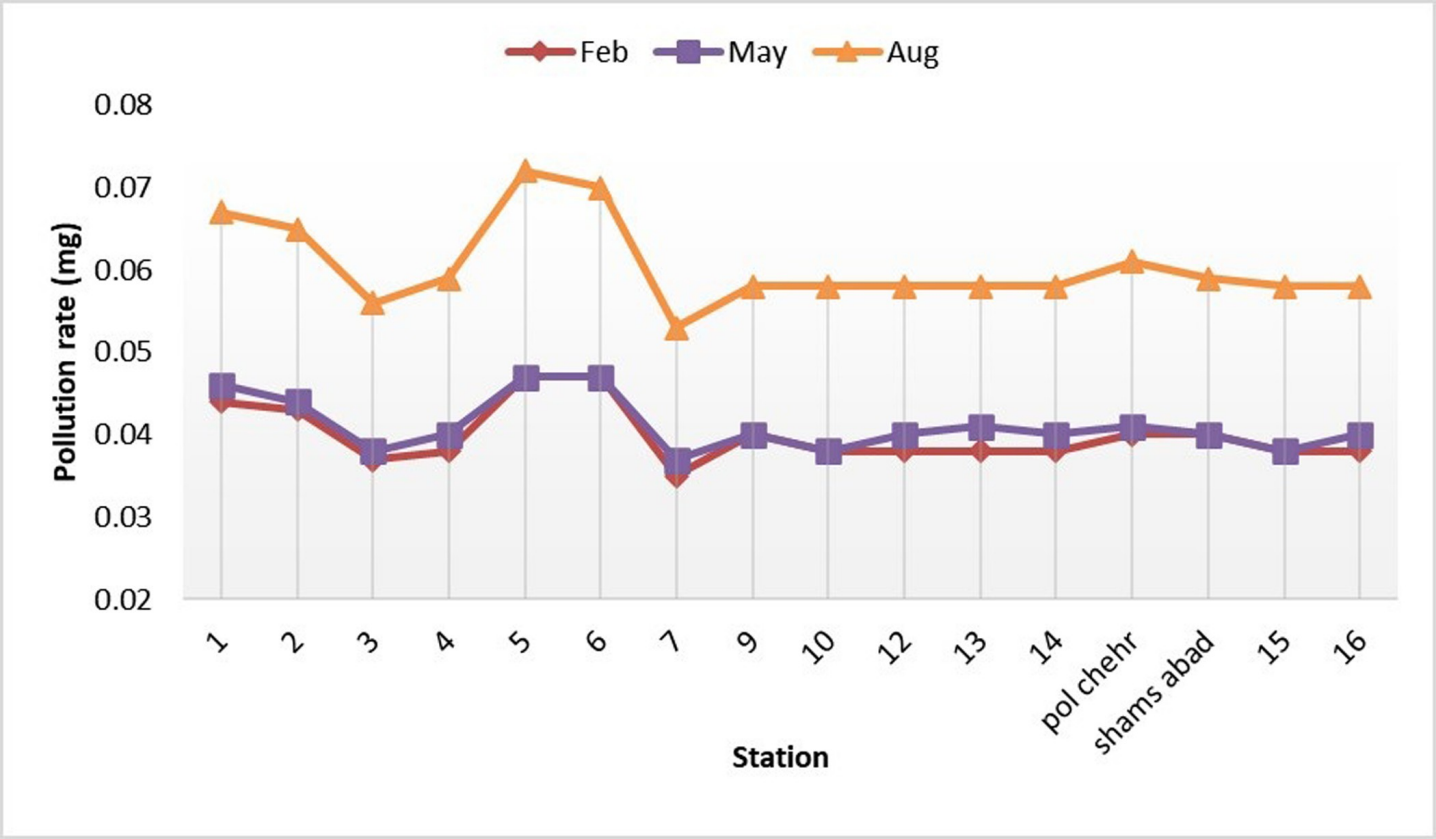
Changes in phosphate along the Gamasiab river.

As displayed in Fig 5, August is still the critical month in terms of the amount of phosphate in the river water, while the other pollutants show similar results. The amount of phosphate in the river comes from different sources. Phosphates are used in water treatment and steam boilers, while high amounts can be found in terminal stations such as Station 14 and Bridge 4 due to the activity of the Bistoun Combined Cycle Powerplant. In addition, the phosphates in the fertilizers used on local agricultural lands brought their highest amount to the water surface during strong winds and storms (around stations 1 to 10) and penetrated the water by melting snow in some cases. Household wastewater especially that generated from detergents is regarded as another factor which creates high phosphate levels.

**Fig 6:**
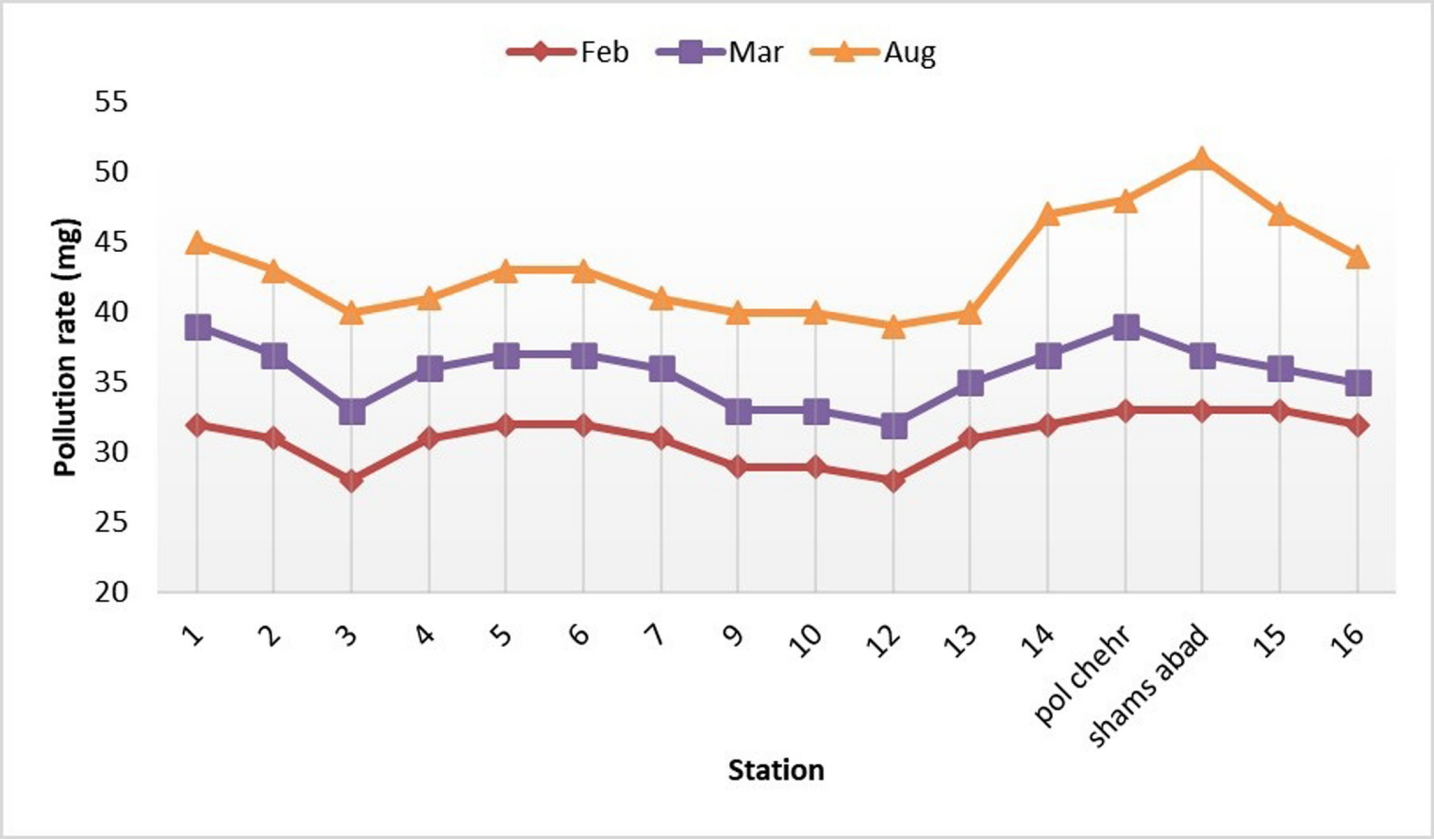
Changes in iron along the Gamasiab river.

Groundwater contains various solutes including iron which is abundantly found in such water. The presence of such solute in water body from the rock beds in the ground with which water comes into contact, resulting in forcing iron to enter the water. The amount of iron concentration in water depends on the duration of time which the water is in contact with such types of stones. Spring and well waters benefit from the highest amount of iron and manganese. However, such solutes cannot be observed by the naked eye. Orange, brown, or black spots quickly form when such waters come into contact with oxygen. Brown and orange spots are created by the iron in the water. Iron in drinking water standards does not pose a risk to human health. However, such element can produce a bitter and metallic taste in water, resulting in creating problems such as staining. Based on the international standards, the amount of iron in water should not exceed 0.3 ppm. Fig 6 illustrates the amount of iron in the sampling stations. Examining the ground structure of the region based on the explanations presented shows that the amount of iron has not experienced considerable change along the way, and there are no visible changes except for the range of stations 14 to 15, which is an industrial area. Therefore, the amount of iron in groundwater water does not change considerably along the way, which is generally in the standard range. However, iron may be higher in other areas due to factors such as industrial pollution.

Figs 3–6 demonstrate that the pollutants measured along the rivers, especially in the dry season, are often at a critical level. However, the amount of iron is an exception and is within the normal range. Water quality time series analysis showed that nitrate, sulfate, phosphate, and iron pollutants have the highest significance in terms of pollution potential, respectively. Such ranking is provided due to the expansion of agriculture in the region, implementation of the tropical plan, delivery of water to agricultural lands, and high consumption of fertilizers to increase land productivity.

### Sulfate pollutant removal

After being transferred to the laboratory, the samples were measured for sulfate according to the existing standard by a DR 5000 spectrophotometer according to Hack, 2013ed8 instructions. The kit includes barium chloride and citric acid as stabilizers. To perform the test, the sample was transferred to a 10 ml tube of the device, and the identifier was added to the sample. The sample was mixed until the identifier was completely dissolved in the sample. After 5 minutes, the sample was rested, and the device was cleaned during this time. To start the work, the Zero button of the device was pressed and placed in the device compartment after cleaning the tube. The results were viewed and recorded by pressing the Read button. The device was placed at a wavelength of 450 nm to determine the sulfate concentration (Salami et al., 2020).

**Fig 7:**
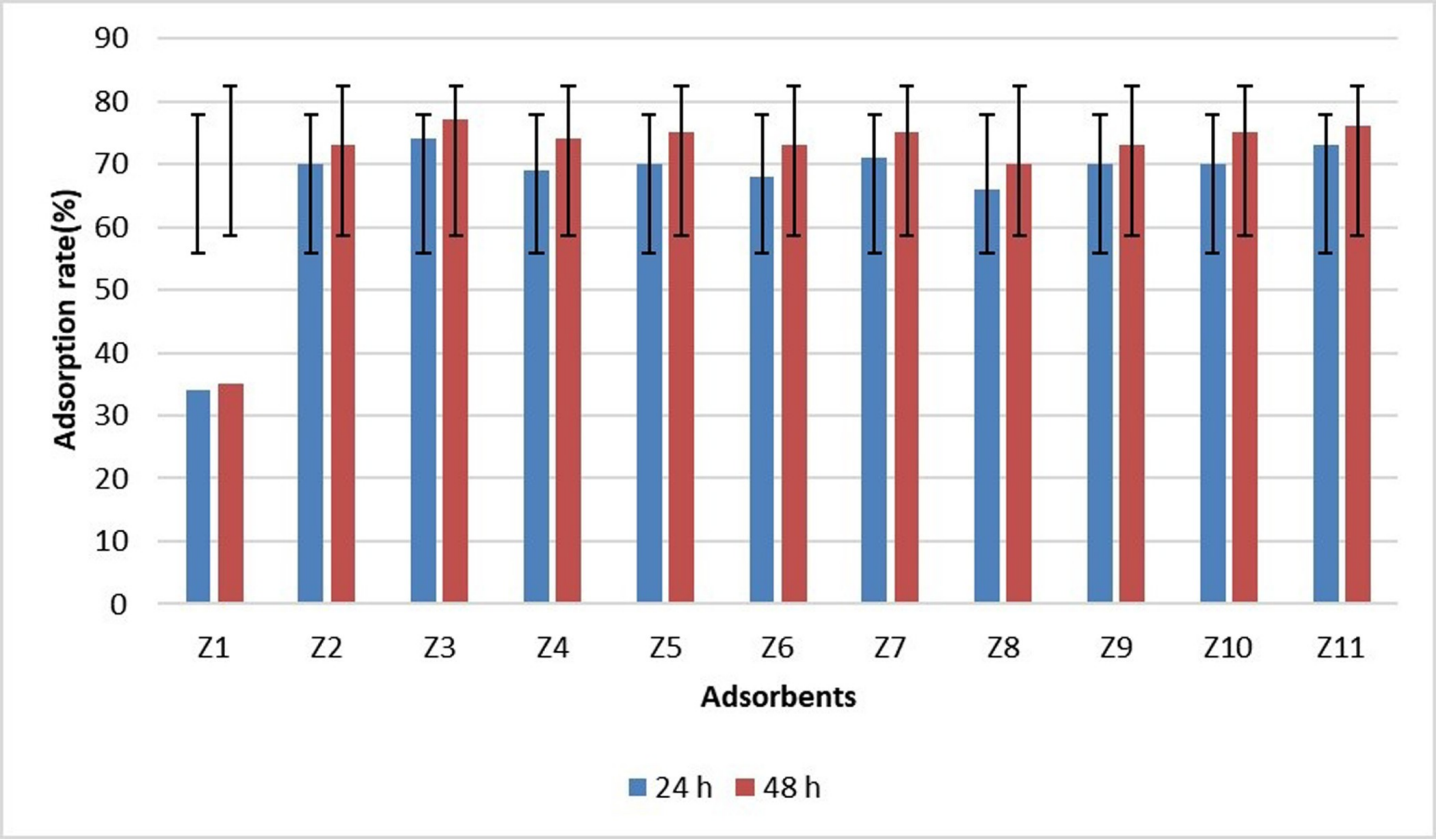
Amount of sulfate after and before purification.

The samples obtained from the measurement during August were selected as the most contaminated samples after calculating the amount of sulfate in the samples (Fig 7). This issue could be predicted due to the reduction of river runoff and use of sulfate-containing fertilizers by farmers at this point. Nanoparticles were added to these samples for sulfate purification, and a surface adsorption mechanism was used for purification. It should be noted that the pH of the samples was set as the pH of the sample taken from the river, and the ratio of adsorbent to pollutant at 25° C for each adsorbent was equal to 5 grams for every 90 cc of 100 mg solution. Fig 7 shows sulfate changes before and after using adsorbents.

The next step involved finding the adsorption equilibrium time. To this aim, the samples were tested for sulfate in specific time steps after applying the adsorbent. These time steps were 30 minutes, 1, 6, 12, 24, and 48 hours after applying the adsorbents [2]. The efficiency was calculated based on Eq (1) after desorption of the nanostructure and measurement of the remaining sulfate in the sample.

Fig 7 illustrates the results of sulfate ion removal by modified adsorbents with different solutions at two times of 24 and 48 hours. The unmodified sample has a removal efficiency of 35%, which increases through different modification methods. As demonstrated, the highest percentage of sulfate ion removal by Z3 adsorbent occurs in 24 hours. The removal of 77% of sulfate within 24 hours and its reduction to 74% after 48 hours shows that the adsorbent reaches its final performance at a high speed and stirring the solution redistributes the pollutant in the water due to the completion of the absorption capacity. This adsorbent was modified by nitric acid and sulfuric acid, both of which are strong acids and improve the holes and impurities, making the adsorbent perform better than other ones.

**Fig 8:**
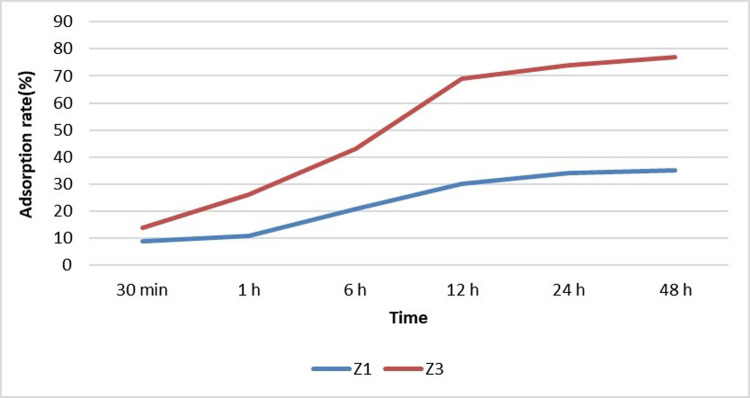
Adsorption rate by time.

Based on the previous studies, 30 minutes, 1, 6, 12, 24, and 48 hours were selected (Fig 8). In the first 30 minutes, the raw sample had less than 30% sulfate ion removal. However, Z3 adsorbent almost increased its removal rate up to 2.5 times compared to the raw sample with the modification of the sample. Over time, the raw sample showed only a 4.2% increase in efficiency until 48 hours later due to the lower absorption power. However, the modified sample displayed a 27% increase in removal efficiency after 24 hours. The upward slope for the modified samples was about the same. Over time, the rising trend of absorption was the same. In this respect, the Z3 sample experienced a decrease in absorption after 24 hours because of reaching the final absorption capacity by the adsorbent. The reason for the relatively low removal efficiency of the adsorbents is that they are not placed in the optimal conditions for their removal including pH, which is considered a neutral environment with an acidity of 7. Another effective parameter is the ratio of the adsorbent to the pollutant, which was set to 5 in this part of the experiment.

### Removing the pollutants interaction

In order to measure the performance of nanoparticles in the adsorption of the target pollutant in the presence of other ions, a solution with a fixed concentration of the target pollutant was prepared based on the points presented in the materials and methods section. Then, different concentrations of anions and cations were added to the solution. Finally, the adsorption process was repeated according to the previous section and the results were presented separately for each pair of ions [27].

### Removing sulfate and phosphate interactions

**Fig 9:**
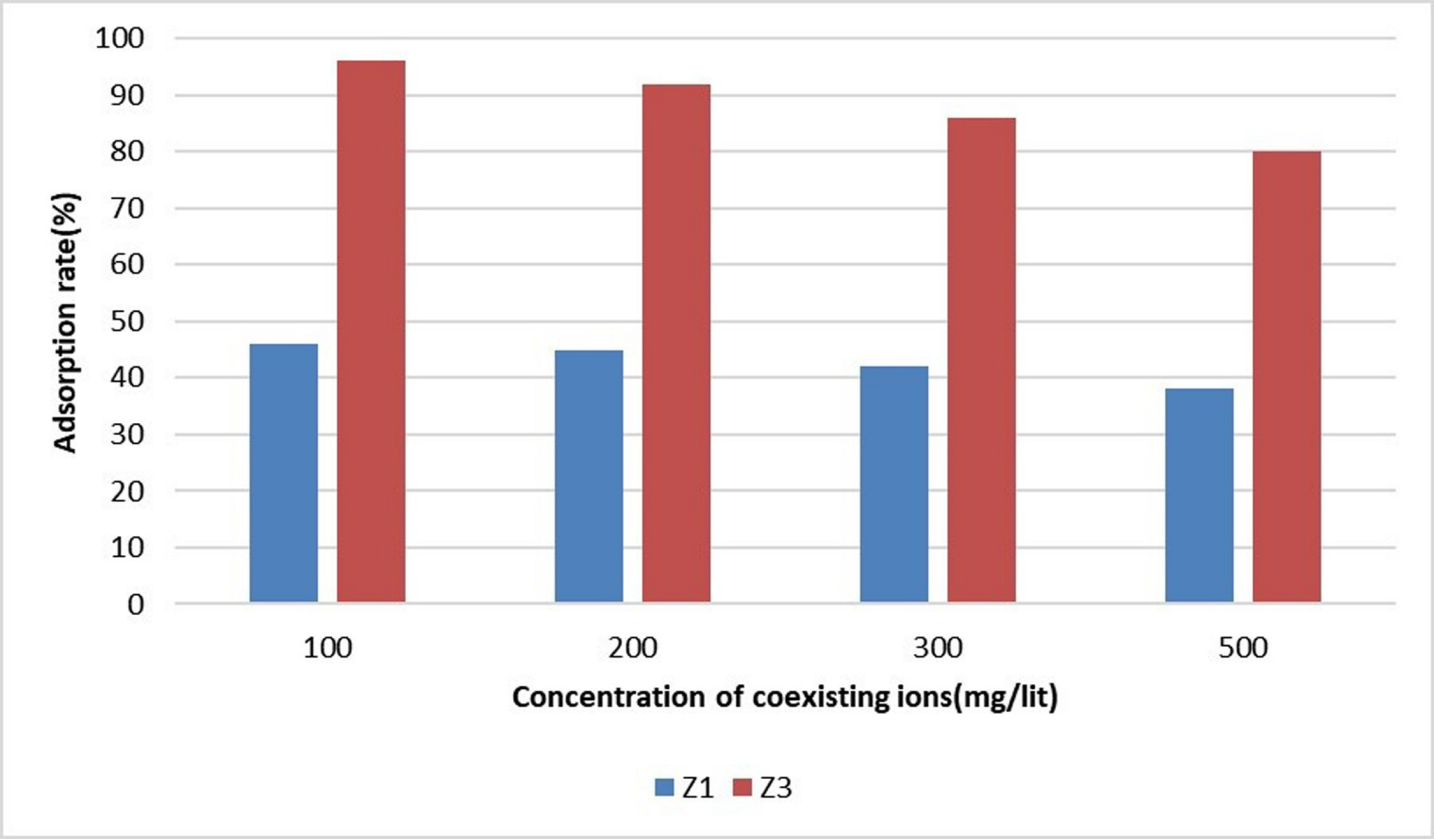
Results of removing sulfate and phosphate interactions.

Other types of anions are generally present in drinking water, which may compete with sulfate ions for the adsorption of zinc in zeolite. To investigate the effect of such interfering ions on sulfate removal, experiments were performed in the presence of 5 mg/liter of sulfate ions and different concentrations of phosphate ions [23]. Phosphate ion concentrations from 100 to 500 mg/liter (100, 200, 300, and 500 mg/liter) were added to the solution, and the adsorption process was repeated. The pH of the solution was equal to that of the river water sample. Fig 9 demonstrates the adsorption results by the superior adsorbent (Z3 and Z1) according to the concentration of coexisting ions.

The competition of the above-mentioned ions is evident in the adsorption process. The sulfate ion adsorption decreases with the increase in concentration although this competition is evaluated at an average level. The results of this section are similar to those reported by Gao et al. [28] who argued that sulfate ion is known as an interfering ion in the phosphate removal process in the removal of nitrate and phosphate.

### Removing sulfate and nitrate interaction

**Fig 10:**
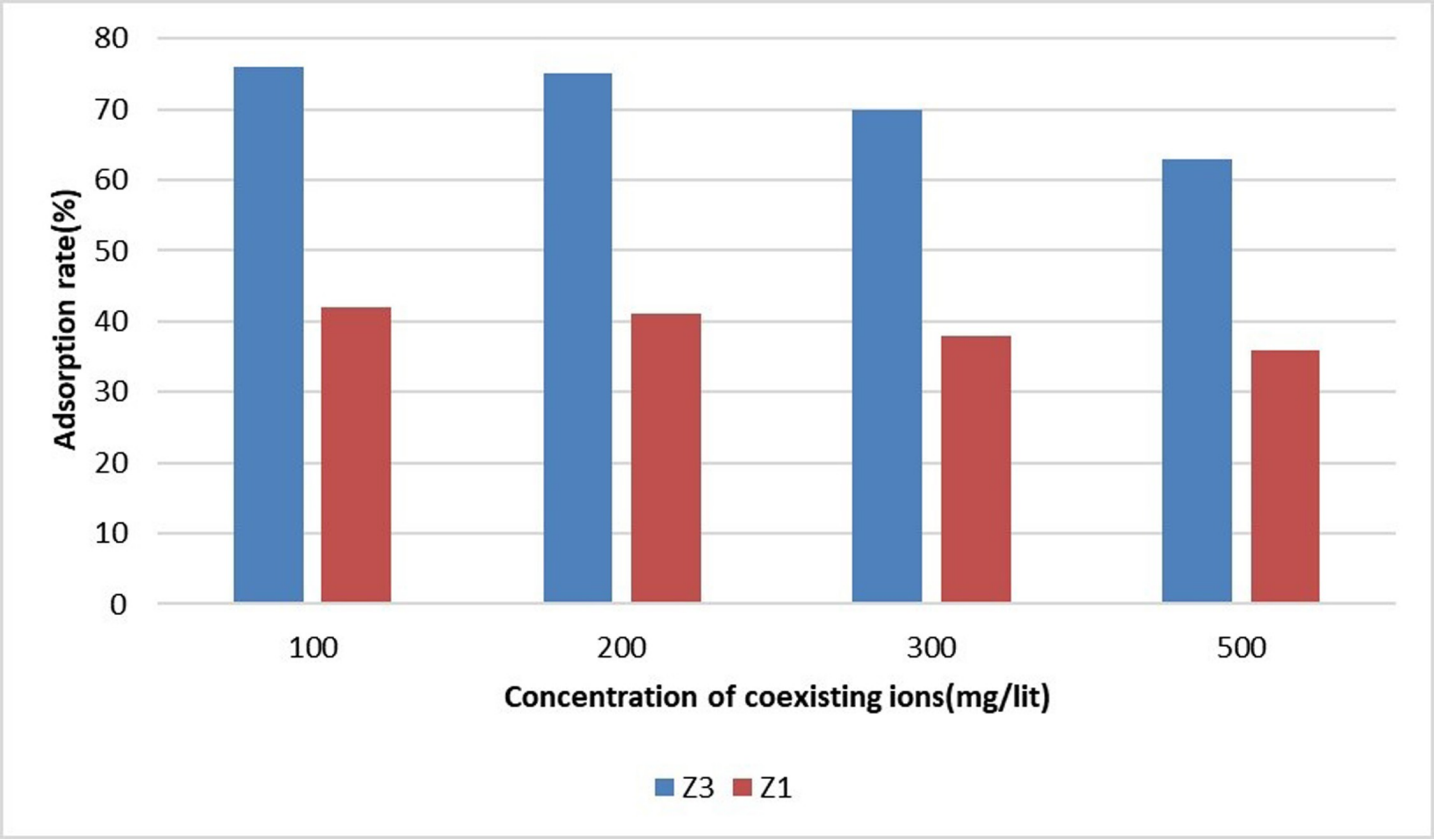
Sulfate and nitrate interaction.

In order to discuss the effect of interfering ions on sulfate removal, the experiments were repeated in the presence of 5 mg/L of sulfate ions and different concentrations of nitrate ions. Nitrate ion concentrations from 100 to 500 mg/L (100, 200, 300, and 500 mg/liter) were added to the solution, and the adsorption process was repeated. The pH of the solution was equal to that of the river water sample. Fig 10 displays the results of adsorption by the superior adsorbents (Z3 and Z1) according to the concentration of coexisting ions 2 hours after the start of the absorption process. Like the interaction of phosphate and sulfate in the adsorption process of nitrate and sulfate, the competition of these two ions was evident. Sulfate ion adsorption decreased with the increase of nitrate ion concentration although this competition was assessed as moderate.

### Removing sulfate and iron interaction

**Fig 11:**
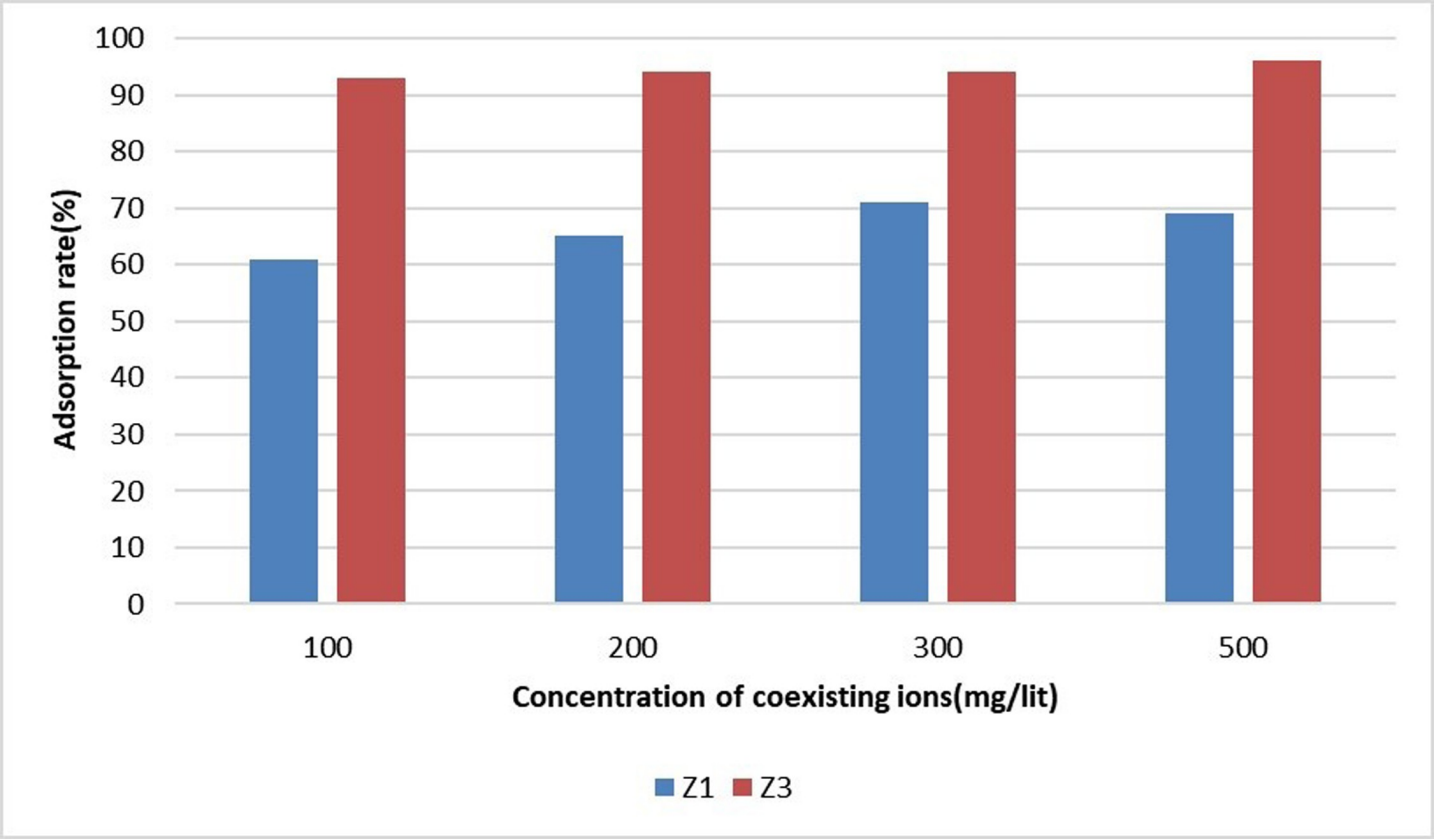
Sulfate and iron interaction.

In order to review the impact of interfering ions on sulfate removal, the experiments were repeated in the presence of 5 mg/L of sulfate ions and different concentrations of coexisting iron ions. Iron ion concentrations from 100 to 500 mg/L (100, 200, 300 and 500 mg/liter) were added to the solution, and the adsorption process was repeated. The pH of the solution was equal to that of the river water sample. Fig 11 shows the results of adsorption by the superior adsorbents (Z3 and Z1) according to the concentration of coexisting ions.

The cooperation of the aforementioned ions was evident in the adsorption process of iron and sulfate. Sulfate ion adsorption increased with the increase of iron ion concentration.

### Experiment design and design expert results for sulfate

The sample removal results indicated that the nanostructure used did not reach its maximum capacity. An economical method should be utilized to achieve its maximum performance. The CCD method is considered as the most critical and comprehensive among surface response methods. Therefore, this method was applied to accurately evaluate the two independent variables. The ratio of the adsorbent (in mg/L) to the initial pollutant concentration (in mg/L) (D/C1) and pH were selected as control variables. Then, the experiment was modeled for the ratio of adsorbent to pollutant and at different pHs employing Design Expert v7.0.0 software.

In the next step, two factors including pH and D/C were selected to analyze the effect of sulfate removal efficiency by the selected adsorbent, Z3. To check the impact of sulfate removal efficiency by the adsorbent, pH and D/C were fed to the software (Table 3) in the range of 3 to 14 and 5 to 100, respectively.

**Table 3:** Experiment design levels and factors for zeolite.

Level	D/C,X_2_	pH,X_1_
-α	5	3
-1	19.1	3.72
0	62.5	7.5
1	86.2	11.68
ɑ	100	14

Table 4 presents the values of pH and D/C factors applied to the software to design the sulfate removal experiment by zeolite.

**Table 4:** Design matrix and laboratory data for effective factors in removing sulfate by zeolite (%).

Experiment No.	Efficiency	Factor	
	Removal percentage	D/C	pH
7	77.1	5	7.5
10	68.4	52.5	7.5
8	52.7	100	7.5
1	69.5	18.91	14
11	68.4	52.5	7.5
9	18.4	42.5	5
3	13.6	86.09	4.32
6	74.8	52.5	12
5	17.1	52.5	3
2	93.3	18.91	10.68
4	65.2	76.1	11.68

In order to assess the parameters, as well as their interaction and squares on sulfate removal by zeolite, the laboratory results can be subjected to variance analysis for different states of sulfate removal. For example, Table 5 represents the results of ANOVA for sulfate removal by zeolite.

**Table 5:** Results of ANOVA for the second-order model of sulfate removal by zeolite from laboratory data.

Source of analysis	Sum of squares	df	Mean of squares	F_value	P_value	State
Model	5666.64	5	1133.33	16.47	0.004	Significant
A_pH	3160.13	1	3160.13	45.92	0.0011	
B_D/C	1696.13	1	1696.73	24.65	0.0042	
AB	222.01	1	222.01	3.23	0.1324	
A^2^	548.59	1	548.59	7.97	0.0370	
B^2^	0.82	1	0.82	0.012	0.9173	
Residual	344.11	5	68.82			
Incompatibility	344.11	3	114.7	-	0.0001<	Significant
Net error	0	2	0			
Modified sum of squares	6010.75	10				

Polynomial and statistically significant models were obtained from the combination of estimates for variables and ANOVA results. These models are given in Eq (5) for sulfate removal by zeolite.


$$\text{Removal}\;\left(\%\right)=+16.07674+17.18832*pH-0.92092*D/C+0.069708*pH*D/C-0.97346*pH2-3.37950E-004*D/C2$$ (5)

The relative error percentage, optimal maximum removal percentage prediction, response level diagram, and balance curve were extracted from Eq (5). In order to check and optimize the sulfate removal model, the laboratory results related to the selected adsorbent were compared with the theoretical model achieved from Eq (5) under identical conditions, resulting in obtaining the average relative error of 4.18% according Table 6. The above-mentioned percentage of relative error is acceptable considering the relative average of the error and presence of two independent and one dependent parameter.

**Table 6:** Validation results of multi-response optimization endpoints for sulfate removal by zeolite.

Sulfate removal efficiency				
Effective Factor pH D/C		Model removal percentage	Library removal percentage	Relative error (%)
4.32	18.91	69.50	60.30	9.20
10.68	18.91	93.30	85.15	8.15
4.32	86.09	13.60	16.27	-2.67
10.68	86.09	67.20	70.92	-3.72
3	52.5	17.10	20.58	-3.48
12	52.5	74.80	76.80	-2.00
7.5	5	77.10	88.23	-11.13
7.5	100	52.70	47.04	5.66
7.5	52.5	68.40	68.40	0.000
7.5	52.5	68.40	68.40	0.000
7.5	52.5	68.40	68.40	0.000
Mean relative error				4.18

**Fig 12:**
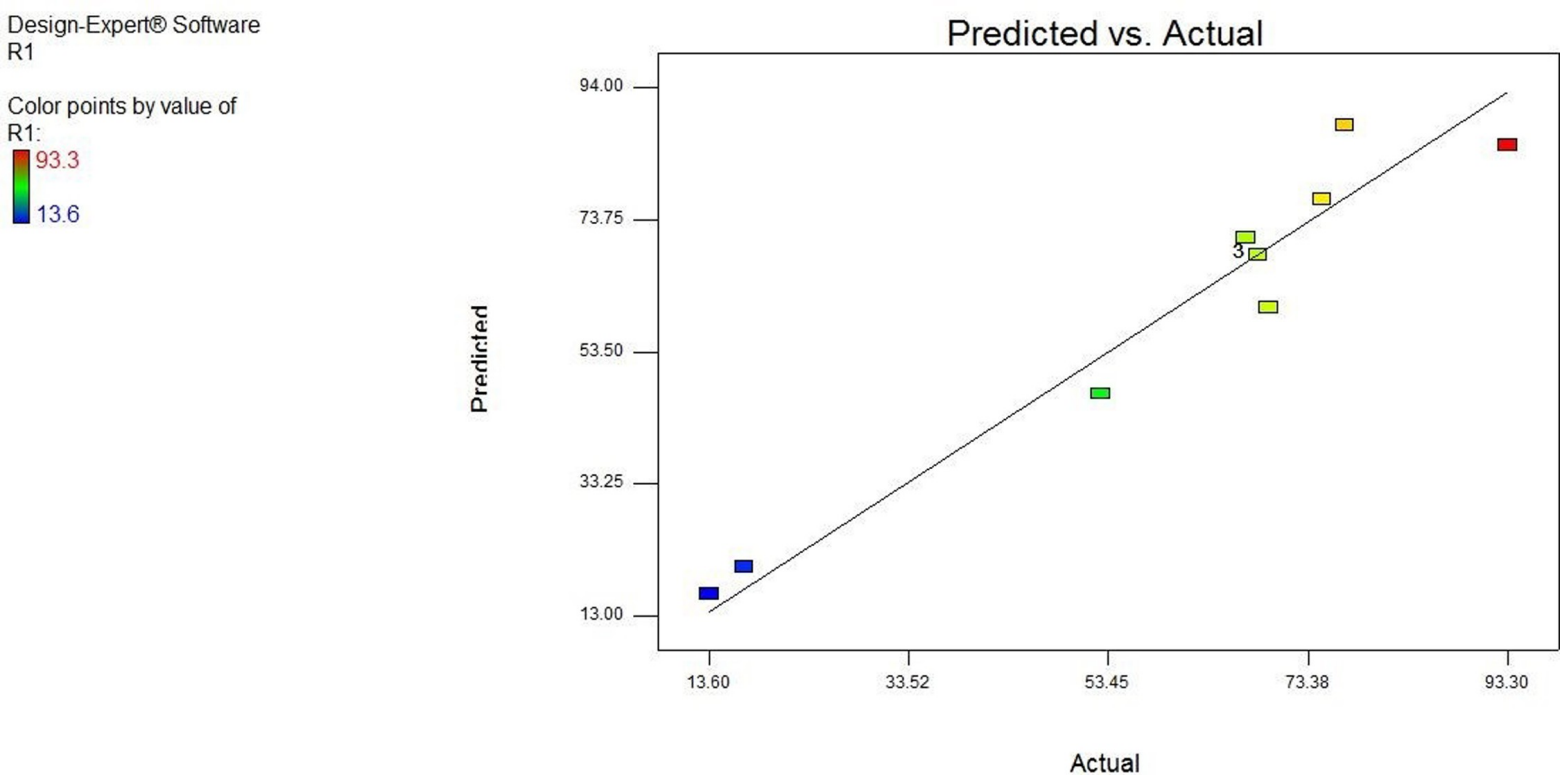
Real and predicted data for zeolite.

**Fig 13:**
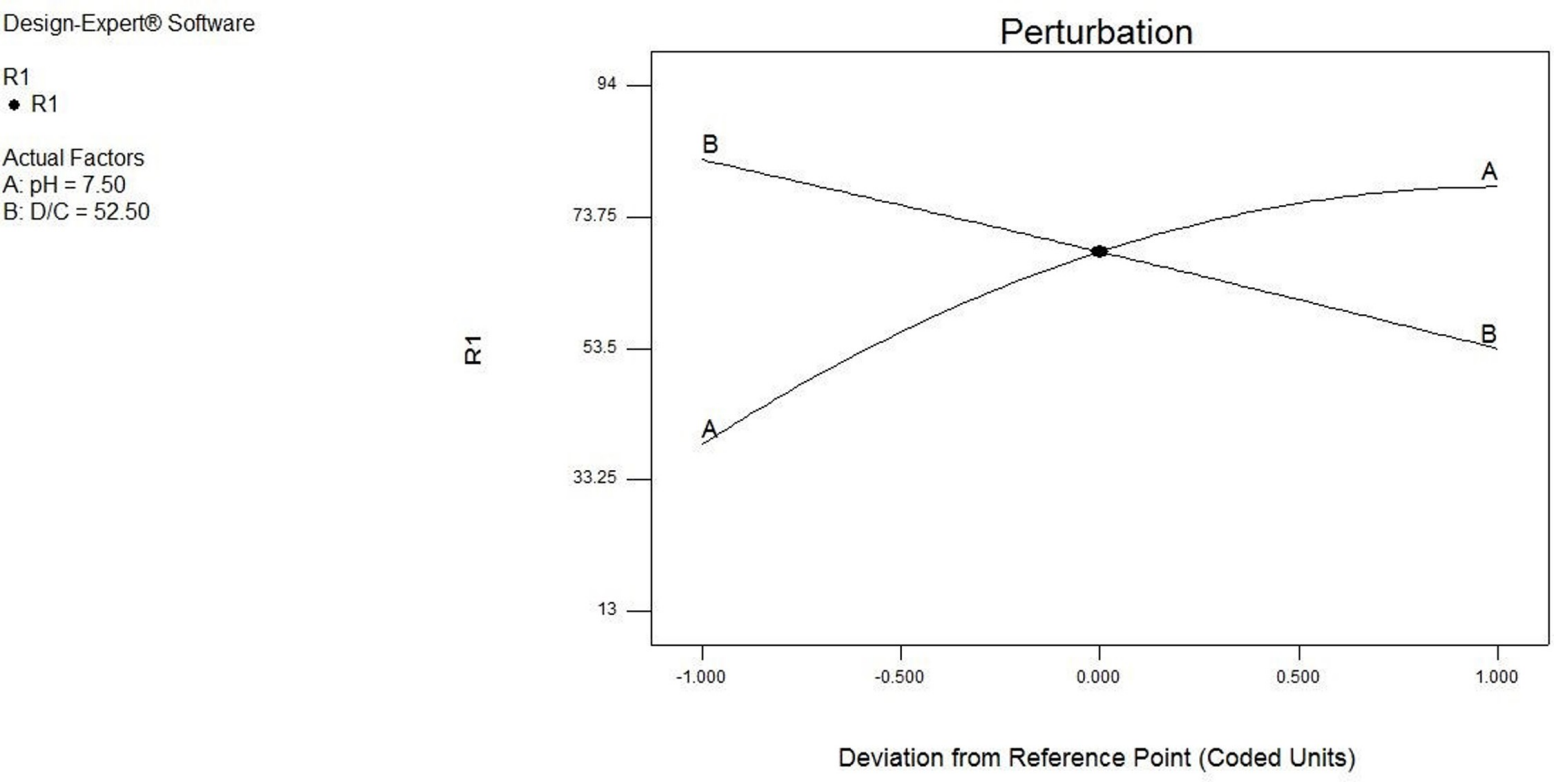
Disturbance curves of the effect of main parameters on sulfate removal by zeolite.

Fig 12 displays the results related to the laboratory model and data for sulfate removal by zeolite. As shown, the closeness of the resulting values to the X = Y line increases the ability of the model to estimate the laboratory results more accurately. As illustrated in Fig 13, the increase in the D/C ratio does not always raise the adsorption percentage. A downward trend was observed for the entire studied range when the aforementioned ratio was selected in the range of 5 to 100. In addition, elimination in the game environment was accompanied by an increase.

**Fig 14:**
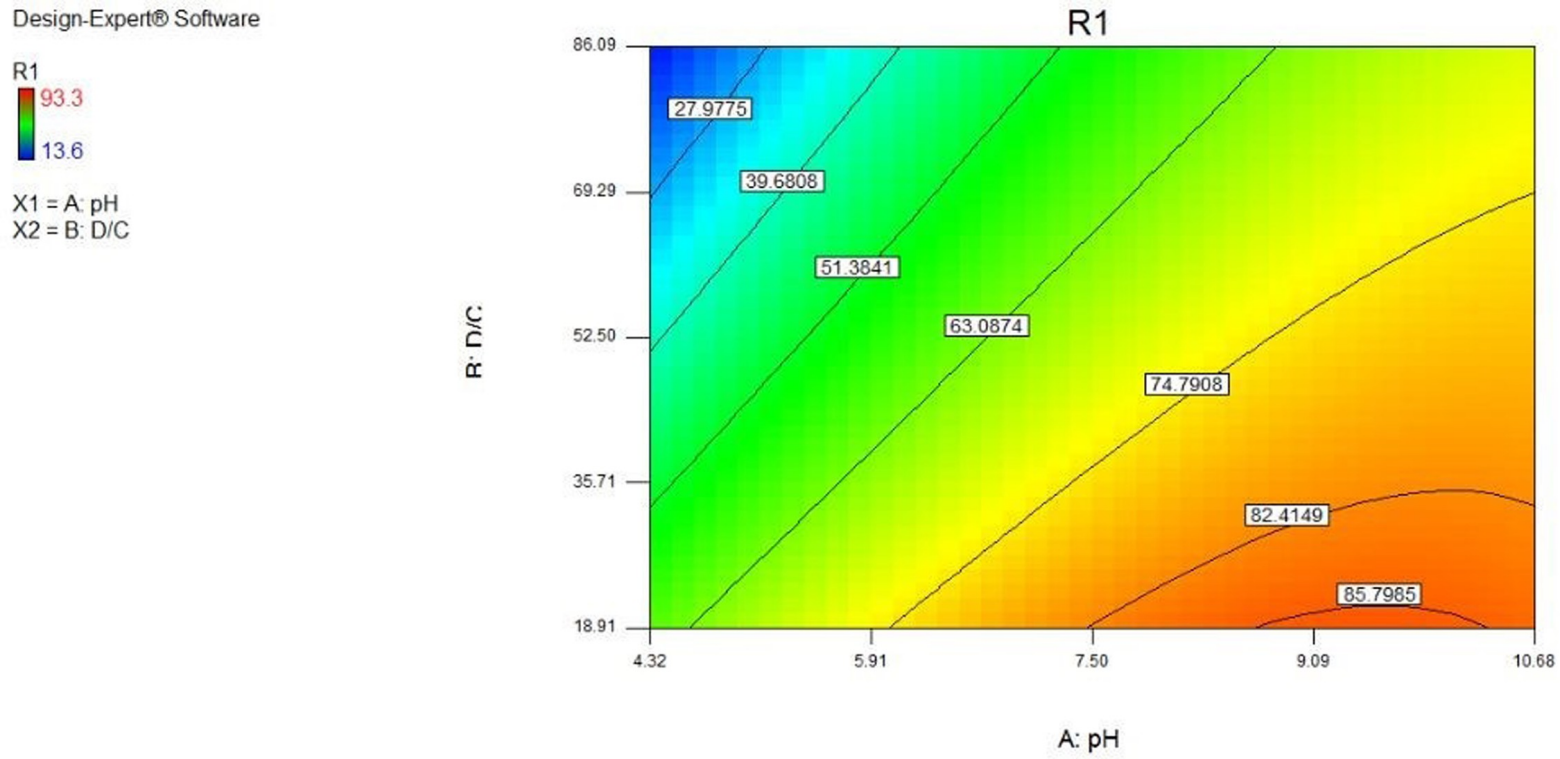
Effect of pH and D/C on sulfate removal percentage by zeolite in the balance curve diagram.

Fig 14 illustrates the simultaneous effect of two factors on sulfate removal. According to the contours drawn in the environment with low acidity (acidic) and high acidity (alkaline environment) compared to the neutral environment, the adsorption efficiency increases when the concentration ratio of adsorbent to the pollutant rises.

**Fig 15:**
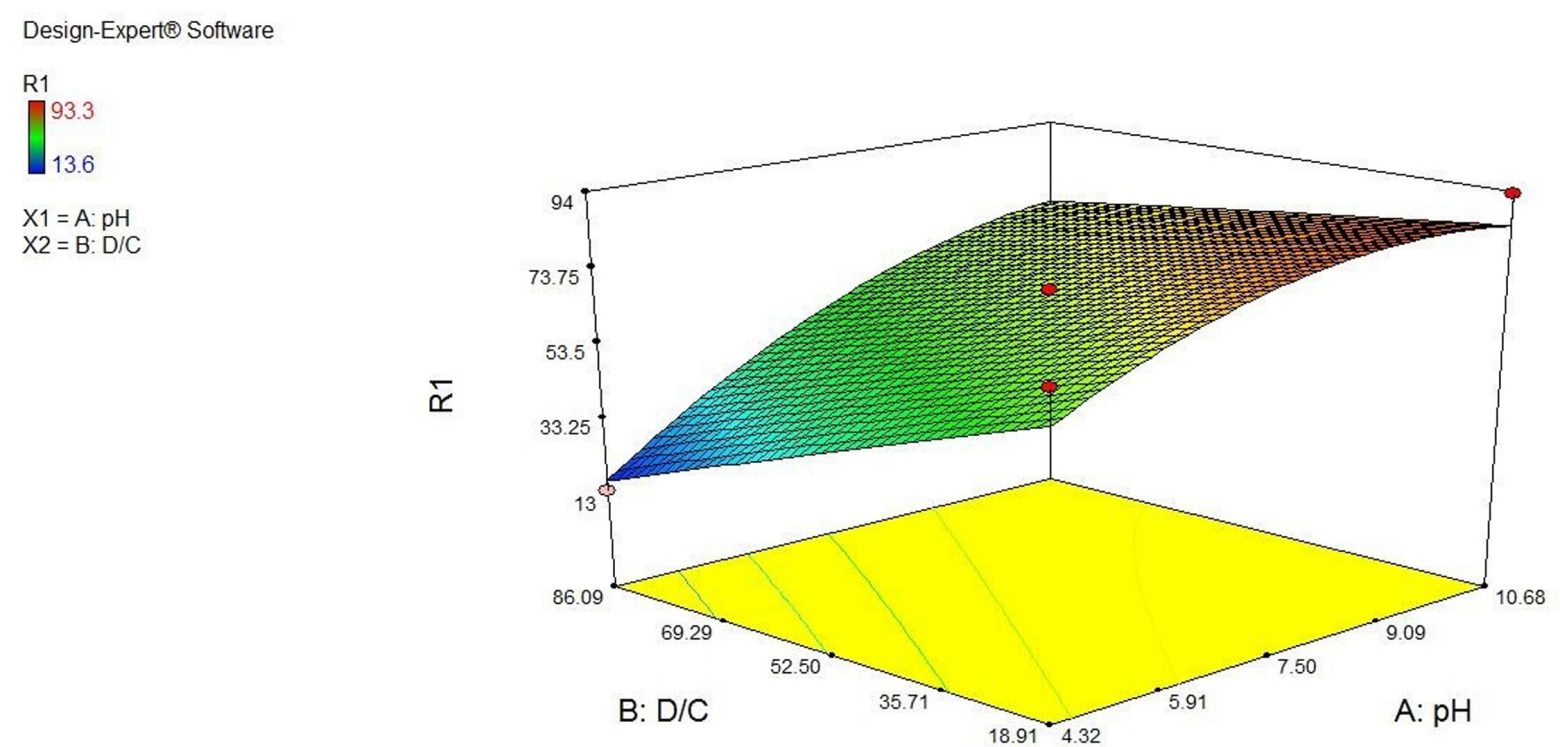
Three-dimensional (3D) diagram of sulfate ion removal percentage by zeolite by surface response method and influencing factors of pH and D/C.

Fig 15 demonstrates that the highest efficiency increases in the game environment. In addition, the removal efficiency increases with the rise in the D/C ratio.

Table 7 shows the points where the optimal removal percentage is obtained with pH and D/C factors. For points with influential factors, this table represents the maximum removal and its appropriateness, which can be observed as a confidence factor for creating conditions to achieve optimal removal.

**Table 7:** Optimal maximum sulfate removal by zeolite points predicted in the software.

No.	pH	Utility	D/C	Removal percentage	
1	9.6	0.92	17.01	89.3	Selection
2	10.5	0.89	17.01	84.233	

### Desorption of adsorbents

Desorption is performed to separate the nanoparticles from the solution and retest by spectrophotometer to determine the amount of the remaining pollutant after applying the adsorbent. In order to desorb zeolite, the filtration method was used by employing Whatman filter paper with a hole size of 10 nm [10].

### Adsorption isotherm

**Fig 16:**
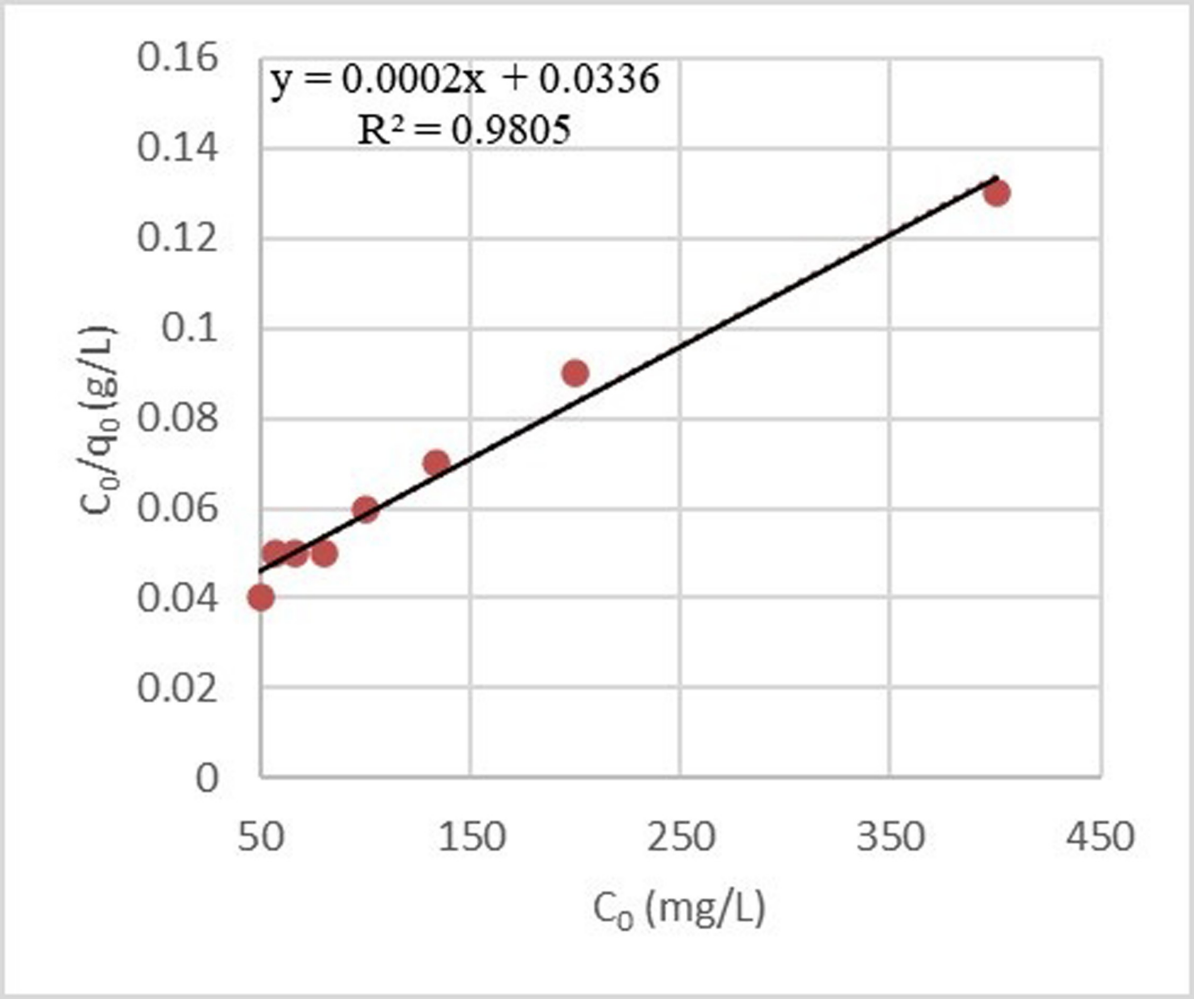
Langmuir model for sulfate adsorption process by zeolite.

**Fig 17:**
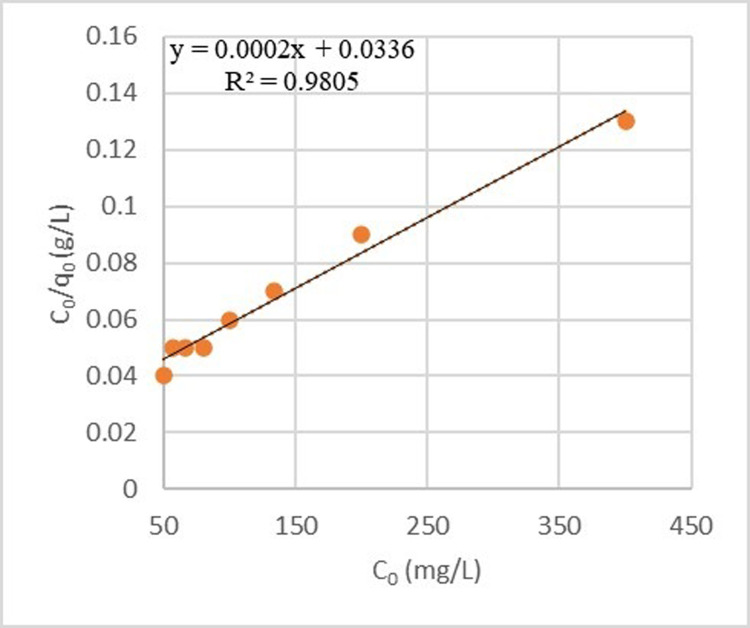
Freundlich model for sulfate adsorption process by zeolite.

Langmuir and Freundlich’s isotherms were calculated for both adsorbents (Figs 16 and 17). As demonstrated, the Langmuir isotherm does not match well with the process of sulfate adsorption by zeolite. The correlation coefficient of the graph with the laboratory data was 0.9805, and this model calculated the theoretical sulfate adsorption capacity to be 74.63 mg/g. The R_L_ coefficient for this pollutant was 0.213. It provided another confirmation for the adsorption process of this ion since it was between zero and one. In addition, the Freundlich isotherm described the sulfate adsorption process by zeolite well. Its correlation coefficient and coefficient of 1/n were 0.9611and 0.1195, respectively.

## Discussion

In the present study, various modifications of zeolite were utilized to adsorb sulfate in a natural water sample collected from the Gamasiab River. The interaction between ions was then investigated. Based on the resulting efficiency, the sulfate amount was measured, and the experiment was designed considering the ratio of D/C and pH. Optimal adsorption points were estimated using the experimental design by altering the pH and the ratio of adsorbent to pollutants in the sample.

The results indicated that unmodified zeolite could remove sulfate with 35% efficiency. In contrast, the adsorption efficiency of modified zeolite to eliminate sulfate was calculated as 77% and 74% using a 0.2 molar solution of nitric acid and sulfuric acid, equilibrium times of 24 and 48 hours, and a D/C ratio equal to 5 grams of adsorbent in 90 cc of 100 mg/L pollutant solution. It was observed that zeolite purification efficiency decreases over time due to reaching its maximum capacity and speed.

To mitigate sulfate, nitrate, and phosphate pollution in river water, appropriate measures should include optimal control and management of construction activities near the river, balanced use of nitrogen and phosphorus-containing fertilizers, efficient management of water resources, and strategic planning to minimize changes in water flow and prevent rainfall interruptions. Similarly, to reduce nitrate pollution, it is essential to control and decrease nitrogen-based fertilizers, implement precise irrigation techniques, adjust agricultural practices, and enhance land management. To address phosphate pollution, optimal water and wastewater treatment methods should be employed, the use of phosphate-containing fertilizers in agricultural lands should be regulated, agricultural techniques should be improved to reduce pollution, and proper wastewater management systems should be optimally applied.

The study revealed that the adsorption of sulfate ions decreases with an increase in phosphate ion concentration, and the competition between these ions was moderate. The adsorption process of sulfate and nitrate resembled the interaction of sulfate and phosphate. Additionally, the adsorption of sulfate ions reduced as the nitrate ion concentration increased, and there was a moderate competition between these ions. Conversely, the interaction of sulfate and iron ions indicated cooperation, leading to an increase in sulfate ion adsorption with higher iron ion concentrations.

Iron enhances sulfate adsorption in water purification by zeolite due to its ability to form strong bonds with sulfate ions. When iron is incorporated into the zeolite structure or used as a coating on the zeolite surface, it can interact with sulfate ions through various mechanisms such as chemical bonding, complexation, or ion exchange. The presence of iron increases the surface reactivity of the zeolite material, providing more active sites for sulfate adsorption. Iron ions can attract sulfate ions through electrostatic interactions and promote the formation of stable complexes, leading to improved adsorption efficiency. Overall, the incorporation of iron into zeolite-based materials enhances sulfate adsorption in water purification processes by improving the adsorption capacity, efficiency, and selectivity of the zeolite towards sulfate ions.

The competition between nitrate and sulfate ions was evident, and the adsorption of sulfate ions decreased with rising nitrate ion concentration. Moreover, the cooperation between iron and sulfate ions during the adsorption process was observed, resulting in an increase in sulfate ion adsorption with higher iron ion concentrations.

The findings align with previous studies (e.g., [28,29]) and suggest that for environmentally sustainable treatment processes, advancements in material development tailored to address specific environmental challenges are crucial. Designing materials that consider local contexts and prevalent pollutants can facilitate appropriate solutions. Research and innovation are essential in materials science to develop multifunctional materials for effective pollutant removal. Emphasizing the intelligent design and application of materials is key to providing targeted, efficient, and eco-friendly solutions for diverse environmental challenges.

By analyzing the effect of pH and D/C on sulfate removal efficiency by adsorbents, the study determined that the optimal points for zeolite adsorption were 9.51 and 18.91, resulting in 86.5% adsorption in the samples. The Freundlich isotherm was found to better match the zeolite purification process for sulfate adsorption compared to the Langmuir isotherm. The study proposes a cost-effective and sustainable approach for water resources using zeolite nanoparticles to address water resource issues in various sectors.

In conclusion, the adsorption methods outlined show the potential for pollutant removal from water, with various common adsorbents such as activated carbon, activated alumina, ionic resins, metal oxides and hydroxides, carbonates, and clays being crucial in water and wastewater treatment. Understanding the adsorption mechanisms through techniques like X-ray diffraction and Fourier-transform infrared spectroscopy is vital for optimizing adsorption performance based on the adsorbent’s physical and chemical characteristics. The adsorption capacity of adsorbents is influenced by their functional groups, such as hydroxyl and carbonate, which play a significant role in removal efficiency [30].

Moreover, pH concentration plays a key role in adsorption capacity by controlling the surface charge of the adsorbent. The type of adsorbent, such as Allophane andisol, can exhibit positive or negative charges depending on the pH range, affecting sulfate removal and acid rain mitigation. The study highlights the importance of understanding the adsorption isotherms of these compounds using models like Langmuir and Freundlich [27].

## Conclusion

This study demonstrates the effectiveness of modified zeolite in adsorbing sulfate from natural water samples, specifically from the Gamasiab River. The findings reveal that while unmodified zeolite achieves a sulfate removal efficiency of 35%, modifications using nitric and sulfuric acid significantly enhance this efficiency to 77% and 74%, respectively. These improvements are attributed to optimal conditions, including a specific adsorbent-to-pollutant ratio and controlled pH levels.

The research highlights the competitive interactions between sulfate, nitrate, and phosphate ions during the adsorption process. Notably, an increase in phosphate concentration was found to decrease sulfate adsorption, indicating moderate competition between these ions. Conversely, the presence of iron ions was shown to cooperate with sulfate ions, enhancing their adsorption through mechanisms such as chemical bonding and complexation. This underscores the potential of incorporating iron into zeolite structures to improve sulfate removal efficiency in water purification applications.

Furthermore, the study emphasizes the importance of sustainable environmental management practices to mitigate pollution in river systems. Recommendations include careful regulation of agricultural practices, optimized use of fertilizers, and improved wastewater management strategies. By addressing these factors, we can minimize the introduction of pollutants like nitrate and phosphate into water bodies. The results align with previous studies and reinforce the need for continued research into advanced materials tailored for specific environmental challenges. Understanding the adsorption mechanisms through various analytical techniques is crucial for optimizing performance based on the physical and chemical characteristics of adsorbents. The study also highlights the significance of pH in influencing adsorption capacity and the role of functional groups in enhancing removal efficiency. In conclusion, this research not only contributes valuable insights into sulfate removal using modified zeolite but also advocates for innovative approaches in material development for effective pollutant management.

## Supporting information

S1 File More information about identifying adsorbents are presented in the supplementary file. In the file, S1 Fig., S2 Fig. and S3 Fig. show identifying zeolite adsorbent using XRD pattern, FT-IR spectrum and TEM image, respectively.
(DOCX)
