# Supplementary material for: Removal of sulfate pollutant from different samples of a river water using nanozeolite technology, case study: Gamasiab River, Iran
Source: PLoS One. 2025 Feb 10;20(2):e0314480. doi: 10.1371/journal.pone.0314480 (PMC11809877; doi:10.1371/journal.pone.0314480)
Supplement: S1 File — In the file, S1 Fig., S2 Fig. and S3 Fig. show identifying zeolite adsorbent using XRD pattern, FT-IR spectrum and TEM image, respectively. (DOCX) [file pone.0314480.s001.docx]

**Supplementary**

***Identifying adsorbents***

Fourier transform impulse range (FT-IR) and transmission electron microscopy (TEM) were achieved from the adsorbent used to identify the characteristics of the adsorbers, X-ray diffraction (XRD) pattern. S1 Fig. displays the XRD pattern for zeolite in the range of angle 10-40° = 2Ɵ. The XRD pattern data show that the utilized stone contains zeolite as the main component and a small amount of quartz (4.5%) and cristobalite (9.2%) phases, and the characteristic lines at 2Ɵ degrees equal to 10, 11.4, 17.4, 23, 26, 28.2, 30, and 32° are similar to the XRD pattern data of zeolite corresponding to JCPDS 0237-38 [10].

S2 Fig. illustrates the FT-IR spectrum for zeolite reached 4000 cm-1. It is worth noting that the measurement was performed applying the in-situ method on pure KBr powder under nitrogen flow. S3 Fig. demonstrates the dispersion of the particles and average size of the particles around 50 nm.

The specifications and explanations related to the results of XRD, FT-IR and TEM analyses are presented. S2 Fig. displays the pattern of chemical connections in the adsorbent, which was measured employing the in-situ method on pure KBr powder under nitrogen flow. S3 Fig. shows that the adsorbent particles are about 50 nm on average. Such microscopic images play a critical role in displaying and analyzing the internal structure of the adsorbent and provide valuable information about the distribution and size of the particles.

XRD analysis showed that the adsorbent contains zeolite as the main component and small amounts of quartz and cristobalite phases. In addition, the FT-IR spectrum indicates that the adsorbent benefits from a specific chemical bonding pattern. Further, the TEM image displays the particle distribution and average particle size of the adsorbent particles. All of these methods and analyses allow the reader to understand the properties of the adsorbent and review its ability to purify and remove pollutants in river water. Such information can help develop optimal water purification methods and reduce river pollution.

| 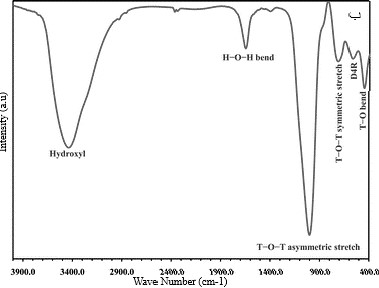 | 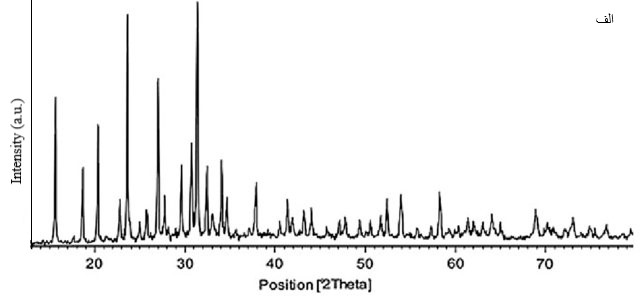 |
| --- | --- |
| **S2 Fig:** Identifying zeolite adsorbent using the FT-IR spectrum | **S1 Fig**: Identifying zeolite adsorbent using XRD pattern |
| 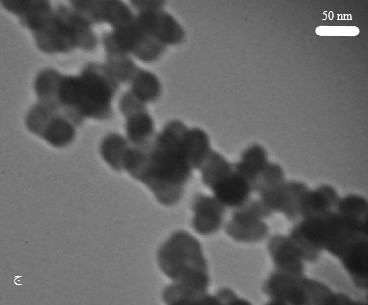 | |
| **S3 Fig.** Identifying zeolite adsorbent using TEM image | |
